# A machine learning framework for precision dosing of daptomycin across diverse disease states

**DOI:** 10.1128/aac.01089-26

**Published:** 2026-10-14

**Authors:** Xin-Fang Zhang, Jing Sun, Guo-Xiang Hao, Xiao-Mei Chen, Yue Zhou, Xiao-Jie Wang, John van den Anker, Wei Zhao, De-Qing Sun, Bo-Hao Tang

**Affiliations:** 1 Department of Pharmacy, The Second Qilu Hospital of Shandong University 531675, Jinan, China; 2 Department of Clinical Pharmacy, Key Laboratory of Chemical Biology (Ministry of Education), State Key Laboratory of Discovery and Utilization of Functional Components in Traditional Chinese Medicine, School of Pharmaceutical Sciences, Cheeloo College of Medicine, Institute of Clinical Pharmacology, Shandong University 12589, Jinan, China; 3 Cheeloo College of Medicine, Shandong University 12589, Jinan, China; 4 Department of Critical Care Medicine, Qilu Hospital of Shandong University 91623, Jinan, China; 5 Center for Health Outcomes Research and Delivery Science, Children’s National Hospital, Washington, DC, USA; 6 Department of Pediatrics, George Washington University School of Medicine and Health Sciences, Washington, DC, USA; Providence Portland Medical Center, Portland, Oregon, USA

**Keywords:** machine learning, individual treatment, pharmacometrics, daptomycin

## Abstract

The large pharmacokinetic variability of daptomycin across disease states poses a challenge for individualized dosing. Conventional population pharmacokinetic (PopPK) models are often limited to specific conditions, lacking generalizability. This study used a machine learning (ML) framework to predict daptomycin exposure and guide dose optimization across heterogeneous populations. Nine published PopPK models were used to generate 90,000 simulated profiles across patients with infections and/or patients treated with renal transplantation and/or renal replacement therapy. Seven ML algorithms were trained to predict the 24 h area under the concentration–time curve (AUC_0–24_). Model performance was evaluated using statistical metrics and clinical scenario assessments, and comparison with PopPK models. External validation was conducted with real-world data. CatBoost demonstrated the highest predictive accuracy, identifying dose, creatinine clearance, disease state, and weight as key predictors. The validation cohort comprised 20 patients with infections (501 concentration measurements) and 19 patients receiving renal replacement therapy (513 concentration measurements). In infected patients, the prediction error of AUC_0–24_ was reduced by 60.1% compared with existing PopPK models (root mean square error [RMSE], 141 vs 353 mg·h/L; mean absolute prediction error, 23.5% vs 58.1%). The ML model correctly classified 75.0% of patients into the appropriate clinical AUC categories (<666, 666–939, and >939 mg·h/L), outperforming PopPK models (average overall accuracy: 30.0%). In patients undergoing renal replacement therapy, ML also improved performance (RMSE, 197 vs 218 mg·h/L; overall accuracy, 52.6% vs 31.6%). The ML model demonstrated accurate prediction of steady-state daptomycin AUC in heterogeneous clinical populations and was successfully validated using real-world data. By enabling accurate pre-treatment exposure prediction, it provides a flexible and clinically useful pathway toward precision dosing of daptomycin.

## INTRODUCTION

Daptomycin, a cyclic lipopeptide antibiotic, is approved for the treatment of gram-positive infections such as complicated skin and skin structure infections and infective endocarditis (1, 2). It demonstrates potent bactericidal activity and is particularly effective against multidrug-resistant pathogens, including methicillin-resistant *Staphylococcus aureus* (MRSA) and vancomycin-resistant enterococci (3). Owing to its unique mechanism of action, daptomycin has become indispensable in the management of severe and refractory infections. It is frequently used in patients with severely impaired renal function or undergoing renal replacement therapy. Both situations require careful dose adjustments. It is also widely applied in patients with osteomyelitis, septic arthritis, or hematological malignancy-related infections (4, 5), reflecting its therapeutic importance across diverse patient populations.

The pharmacokinetics of daptomycin exhibit considerable interindividual variability, driven by factors such as body weight, sex, renal function (creatinine clearance, CRCL), and specific disease states (6, 7). This variability complicates the achievement of effective yet safe drug exposure (8, 9), which is critical as daptomycin’s efficacy and toxicity are closely tied to the area under the concentration–time curve (AUC_0–24_) (10–12). Traditionally, disease-specific population pharmacokinetic (PopPK) models have been developed to guide dosing. However, this approach has inherent limitations: the proliferation of models tailored to specific conditions (e.g., one for RRT, one for hematologic malignancies, etc.) fragments clinical knowledge and complicates practical application, as clinicians must first identify the most appropriate model for a given patient. Moreover, the technical expertise required to implement these models limits their accessibility in clinical practice.

To overcome the limitations of fragmented, disease-specific models, we hypothesized that a unified framework could be built by integrating existing PopPK knowledge. Machine learning (ML) is uniquely suited for this task, as it can efficiently learn from large, heterogeneous data sets and uncover complex, nonlinear relationships without pre-specified structural assumptions.

Therefore, this study aimed to develop and validate an ML-based framework that integrates multiple published PopPK models to predict daptomycin exposure (AUC_0–24_) across a spectrum of disease states. We sought to create a single, robust tool that transcends the boundaries of traditional models, ultimately providing a more generalizable and practical strategy for the precision dosing of daptomycin in diverse clinical populations.

## MATERIALS AND METHODS

### Data set construction

**Fig 1:**
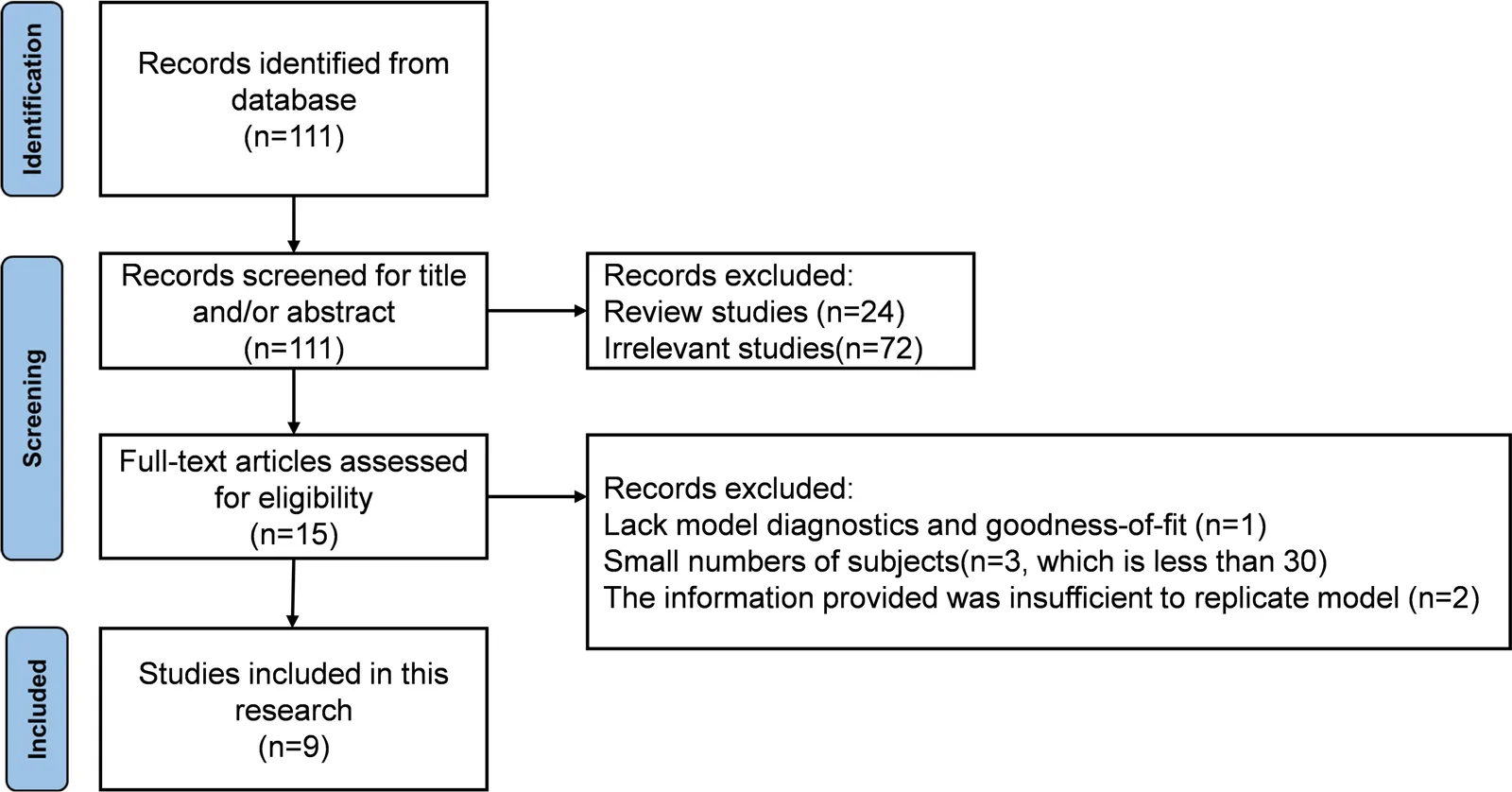
The PRISMA flow diagram for study identification.

A systematic literature search was conducted through the PubMed journal database to search for published PopPK models of daptomycin. Search terms included daptomycin, population pharmacokinetics, quantitative pharmacology, and model-informed precision dosing. Studies were excluded if they were irrelevant, lacked model diagnostics and goodness-of-fit, had a small sample size (*n* < 30), or provided insufficient information for model replication. The selection process is summarized in the PRISMA flow diagram (Fig. 1). Ultimately, nine validated PopPK studies were retained, encompassing renal transplant recipients, patients receiving renal replacement therapy, individuals with renal impairment, and patients with various infections (MRSA, bone and joint infections, and severe infections) (9, 13–20). The characteristics of the eight selected studies are listed in Table 1.

**TABLE 1:** Characteristics of the nine selected studies^*a*^

Model	Author	Study population	No subjects	No. observations	Covariate
1	Dvorchik et al. (9)	Healthy and infected	282	3,325	WT, CRCL, TEMP, sex, infection, dialysis
2	Lou et al. (13)	Kidney transplant recipients	49	537	WT, GFR
3	Wu et al. (14)	Critically ill patients	64	737	CRCL
4	Xie et al. (15)	Patients undergoing continuous renal replacement therapy	32	–	WT
5	Garreau et al. (16)	Bone and joint infection	183	1,303	CRCL, sex, age, RIF
6	Balice et al.(17)	Severe gram-positive infections	94	–	CRCL, sex
7	Yamada et al. (18)	Methicillin-resistant *Staphylococcus aureus* infections	47	110	CRCL
8	Di Paolo et al. (19)	Severe gram-positive infections	58	158	CRCL
9	García-Martínez et al. (20)	Patients with renal impairment	46	157	CRCL

^*a*^ WT, weight; TEMP, body temperature; CRCL, creatinine clearance; GFR, glomerular filtration rate; RIF, rifampicin co-administration; ALB, albumin; –, not reported.

A total of 9,000 virtual patients were generated and distributed across the nine models. Continuous covariates were simulated based on distributions derived from our daptomycin clinical database. Body weight was simulated using sex-specific normal distributions (75 ± 12 kg for males, truncated to 40–120 kg; 65 ± 12 kg for females, truncated to 35–100 kg). Age was simulated using a normal distribution (54 ± 10 years), truncated to 18–85 years, and serum albumin (ALB) was simulated using a normal distribution (36 ± 7 g/L), truncated to 12–60 g/L. CRCL was included as a covariate in almost all published PopPK models, which were developed using patient populations with varying renal function. CRCL was simulated using a normal distribution based on the mean and standard deviation reported for the corresponding population in each original PopPK model and was sampled independently of the other covariates. For continuous covariates that could not be realistically simulated, such as body temperature, the reference values defined in the original PopPK models were fixed to eliminate their covariate effects during simulation. For categorical covariates, sex was randomly assigned using a uniform distribution. Other categorical covariates, including dialysis and concomitant rifampicin (RIF) use, were not included because the corresponding subgroups contained fewer than 30 patients in the original PopPK studies. Only exposure profiles representative of the major patient populations in the original models were simulated.

Monte Carlo simulations were conducted using parameter estimates from the PopPK models. Individual PK parameters were generated using the original inter-individual variability; covariance terms were retained when reported in the original models. Residual error was set according to the original PopPK models. Each virtual patient received 10 dosing regimens: 4, 6, 8, 10, or 12 mg/kg administered every 24 or 48 h. All doses were administered as 1 h intravenous infusions. Simulations were performed directly under steady-state conditions, assuming that the treatment duration was sufficient to reach steady state. At steady state, plasma concentrations were simulated at 0.5, 1, 1.5, 2, 3, 4, 8, 12, 18, and 24 h after dosing. In total, 90,000 pharmacokinetic profiles were generated, and AUC_0–24_ values were calculated using the trapezoidal method. For q48h dosage regimens, AUC was calculated as the average 24 h exposure over the 48 h dosing interval.

### ML model construction

**Fig 2:**
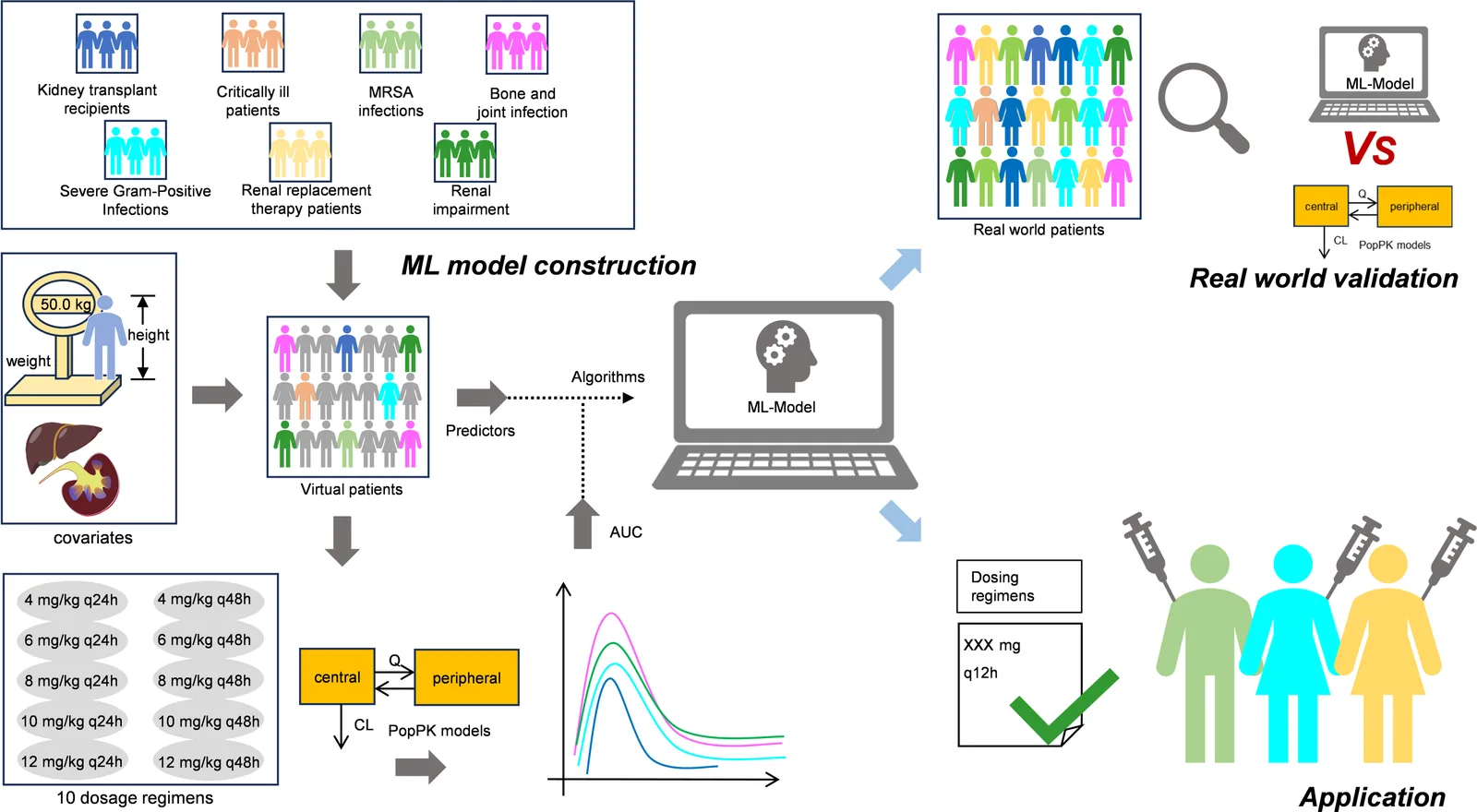
Flowchart of this study.

The simulated data set was randomly divided into the training data set (80.0%) and test data set (20.0%). Predictors included weight, age, sex, ALB, CRCL, dose, and disease states. Disease states were categorized into three groups: (i) infections (MRSA, bone and joint, and severe infections), (ii) renal replacement therapy, and (iii) renal transplantation. Renal impairment was not further categorized as an independent disease state because renal function was quantitatively represented by CRCL as a continuous covariate in the PopPK models. The ML models were trained as regression models to predict AUC_0–24_ values. Seven ML algorithms were evaluated: Random Forest Regressor, Ridge, Lasso, ElasticNet, KNeighbors Regressor, Decision Tree Regressor, and categorical boosting (CatBoost). The flowchart of this study is shown in Fig. 2.

Predictive performance was assessed using root mean square error (RMSE), mean absolute prediction error (MAPE), and mean prediction error (MPE). In addition, prediction error, absolute prediction error, percentage prediction error, and absolute percentage prediction error were summarized as mean ± SD and median (Q1–Q3).


$$\begin{array}{rl} RMSE & =\sqrt{\frac{1}{N}\sum {\left({AUC}_{pred}{-AUC}_{ref}\right)}^{2}}\\ MAPE & =\frac{1}{N}\sum \frac{\left|{AUC}_{pred}-{AUC}_{ref}\right|}{{AUC}_{ref}}\times 100\%\\ MPE & =\frac{1}{N}\sum \left({AUC}_{pred}-{AUC}_{ref}\right) \end{array}$$

where AUC_pred_ represents the predicted AUC_0–24_ value and AUC_ref_ represents the reference AUC_0–24_ value.

Feature importance was further explored using Shapley Additive Explanations (SHAP), implemented in Python, to quantify the contribution of each covariate to model output (21).

### Real-world data validation

To evaluate the predictive capability of the final ML model, real-world data were collected from the intensive care unit of Qilu Hospital, Shandong, China. The validation cohort included patients with severe infections and those undergoing renal replacement therapy. Each subject collected at least five blood samples at 1, 2–4, 4–6, 6–8, 8–12, 12–16, and 16–24 h after daptomycin administration. Individual AUC_0–24_ values (reference values) were calculated directly from measured concentration–time data using non-compartmental analysis based on the trapezoidal rule.

Model validation comprised two levels of evaluation: (i) statistical evaluation—RMSE, PE, and APE were calculated to evaluate predictive performance, with PE and APE summarized as both. mean ± SD and median (Q1–Q3); and (ii) clinical scenario evaluation—reference AUC_0–24_ values were stratified into three clinically relevant categories: <666 mg·h/L, 666–939 mg·h/L, and >939 mg·h/L. ML-predicted AUC values were then compared against observed categories, and classification accuracy was calculated within each range.

### Comparison of prediction performance with PopPK model

A head-to-head comparison was conducted between the ML model and conventional PopPK models. Of the nine PopPK models used for data simulation, five were derived from populations with infections (16–19, 22), and one from patients receiving renal replacement therapy (6). These models were applied to the real-world data set, and their predictive performance was compared with that of the ML model. For both approaches, individual AUC_0–24_ values were predicted a priori using patient covariates and dosing information, without incorporating measured drug concentrations.

For the PopPK approach, individual AUC_0–24_ predictions were generated using typical population parameter estimates, relevant covariates, and dosing information. The residual error was set to an additive error of 0.01 mg/L and a proportional error of 0.01%. Performance metrics were directly compared across methods to determine whether ML provided superior predictive accuracy and generalizability.

## RESULTS

### Data set construction

A total of 90,000 AUC_0–24_ data from 9,000 virtual patients were generated, with each patient receiving 10 dosing regimens. The median AUC_0–24_ was 684 mg·h/L (range, 142.3–3,861 mg·h/L). The median weight was 70.4 kg (range, 42.0–108.8 kg). The characteristics of virtual patients are presented in Table 2.

**TABLE 2:** Summary of simulation characteristics and real-world validation cohorts^*a*^

Median (range)	Simulations (*n* = 9,000)	Real patients—infected patients (*n* = 20)	Real patients—renal replacement therapy patients (*n* = 19)
Age (years)	54.2 (18.04–84.98)	56.5 (11–84)	53 (34–83)
Weight (kg)	70.36 (42.04–108.77)	65 (33–95)	70 (40–92)
ALB (g/L)	36.51 (13.33–48)	34.5 (26–48.4)	37.1 (26–57.7)
GFR (mL/min)	42.30 (10.42–225.29)	69.97 (12.61–184.89)	32.2 (12.67–119.6)
CRCL (mL/min)	48.28 (9.24–287.43)	69.19 (15.98–157.23)	44.61 (16.84–87.35)
Dose (mg/kg)	8 (4–12)	7.69 (5.26–9.09)	7.14 (5.43–12.5)
AUC_0–24_ (mg·h/L)	683.75 (42.39–3,860.87)	549.86 (308.98–1,454.94)	753.83 (397.63–1,125.82)
*C*_min_ (mg/L)	11.93 (0.10-113.55)	9.52 (4.26–42.65)	17.27 (3.57–28.9)
*C*_max_ (mg/L)	81.73 (14.39–343.04)	59 (31.3–87.4)	56.5 (40–98.8)
CL (L/h)	0.589 (0.197–7.08)	0.91 (0.206–1.62)	0.663 (0.444–1.26)
*V* (L)	6.49 (0.95–12.67)	13.2 (7.17–21.93)	14.67 (8.61–32.64)

^*a*^ ALB, albumin; GFR, glomerular filtration rate; CRCL, creatinine clearance.

### ML model construction

Multiple ML algorithms were trained on the simulated data set to predict AUC_0–24_. As summarized in Table 3, CatBoost exhibited the best predictive accuracy and was selected as the final model. CatBoost, an optimized gradient boosting framework, is particularly effective in handling categorical variables and mitigating overfitting (23, 24).

**TABLE 3:** Test set performance measures for all regressors^*a*^

Methods	RMSE (mg·h/L)	R2	MPE (%)	MAPE (%)
RF	303.84	0.644	13.58	31.95
Ridge	239.25	0.779	0.21	29.13
Lasso	239.28	0.779	0.47	28.96
ElasticNet	243.02	0.778	4.95	26.75
KNeighborsRegressor	202.15	0.844	5.01	17.49
DecisionTreeRegressor	285.4	0.685	11.28	28.54
CatBoostRegressor	163.82	0.896	4.3	14.77

^*a*^ RMSE, root mean square error; MPE, mean percentage error; MAPE, mean absolute percentage error.

**Fig 3:**
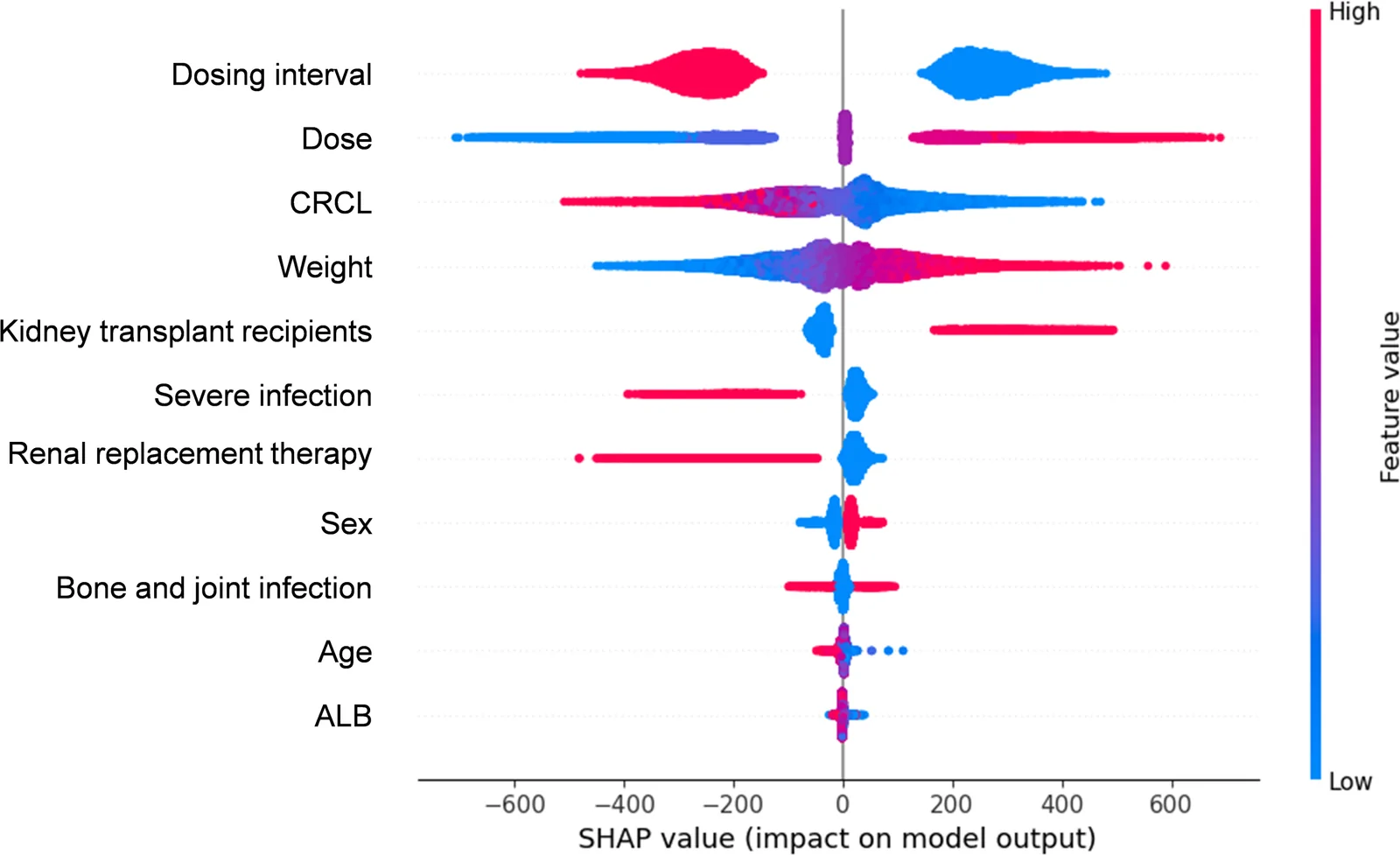
SHAP results of model features. Each point represents a sample. The redder the color, the larger the feature value, and the bluer the color, the smaller the feature value. If most SHAP values of the red points are located in the area greater than 0, and most SHAP values of the blue points are located in the area less than 0, it means that the feature has a positive correlation with the target variable and vice versa. For disease-related binary variables, 1 indicates the presence and 0 indicates the absence of the condition; for sex, 1 indicates male and 2 indicates female.

The SHAP results are shown in Fig. 3, which visualized the contribution of each feature to the model’s output. For the final ML model, dose, CRCL, disease states, weight, ALB, sex, and age were the primary determinants of AUC_0–24_. Dose, weight, and ALB correlated positively with AUC_0–24_. CRCL and dosing intervals were correlated negatively with AUC_0–24_. Disease-specific effects were also observed: patients on renal replacement therapy or severe infection exhibited lower AUC_0–24_ values, whereas renal transplantation showed higher AUC_0–24_. Additionally, females demonstrated slightly greater AUC_0–24_ compared with males.

### Real-world data validation

**Fig 4:**
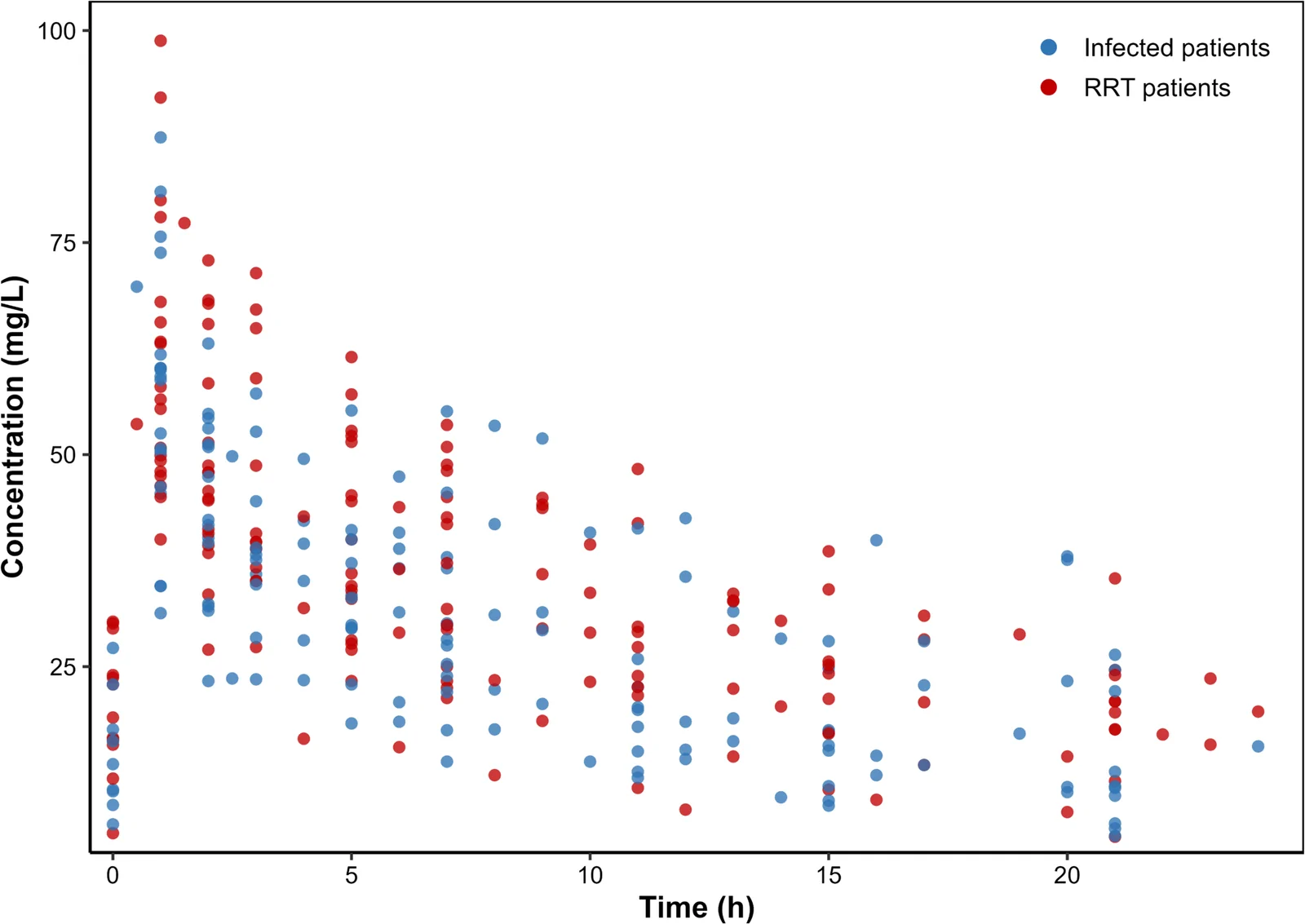
Daptomycin concentration vs time (real-world data).

The validation cohort comprised 20 patients with infections (501 concentration measurements) and 19 patients receiving renal replacement therapy (513 concentration measurements), from which individual AUC_0–24_ values were calculated. The characteristics of real patients are presented in Table 2. The concentration–time profiles of the validation cohort are shown in Fig. 4.

#### Infected patients

**Fig 5:**
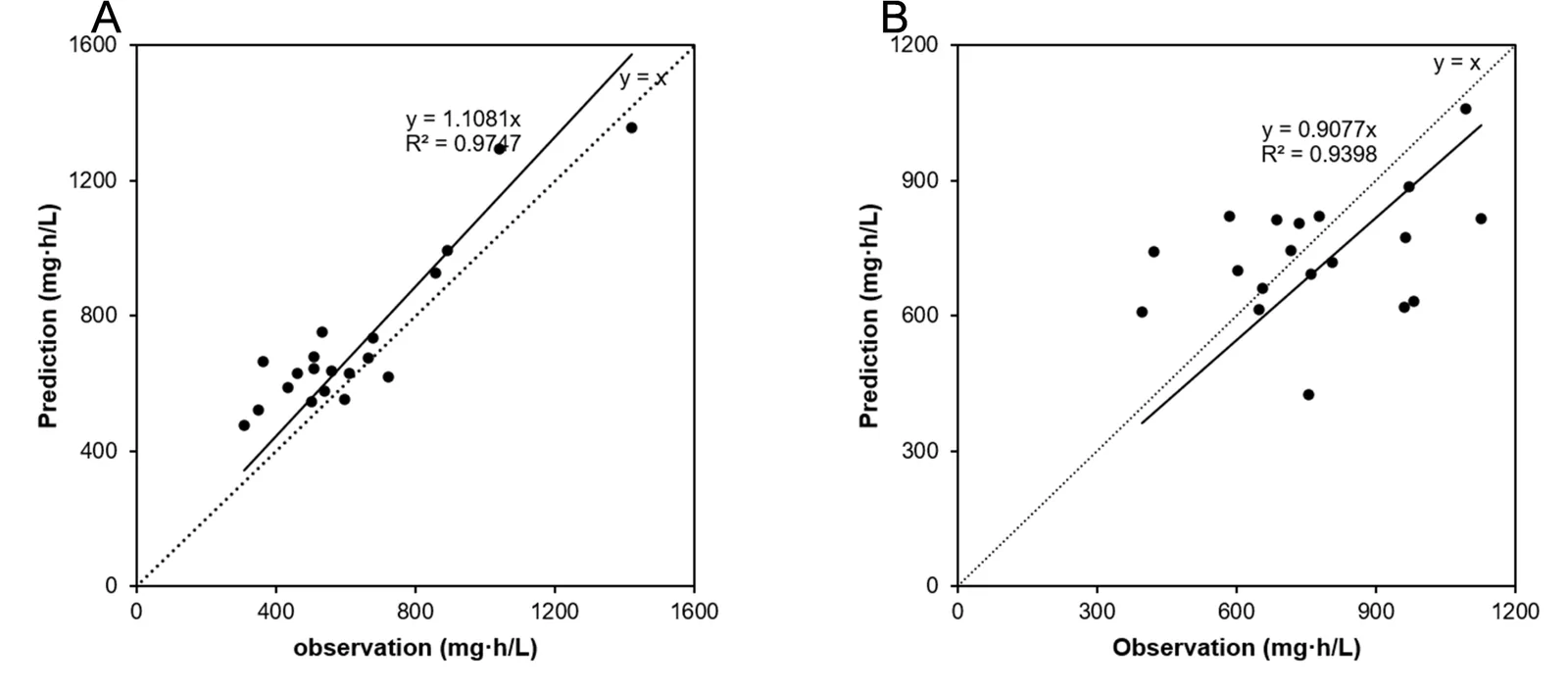
Goodness-of-fit results of the real-world data validation: (**A**) infected patients and (**B**) renal replacement therapy patients.

The final ML model achieved a MAPE of 23.5% and an RMSE of 141 mg·h/L (Table 4). Clinical scenario evaluation demonstrated prediction accuracies of 78.6% (<666 mg·h/L), 50.0% (666–939 mg·h/L), and 100% (>939 mg·h/L), yielding an overall accuracy of 75.0% (Table 5). Goodness-of-fit results of the real-world data validation are shown in Fig. 5.

**TABLE 4:** Comparison of statistical indicators with PopPK models^*a*^

Patient group	Model	RMSE (mg·h/L)	PE (mg·h/L), mean ± SD	PE (mg·h/L), median (Q1–Q3)	APE (%), mean ± SD	APE%, median (Q1–Q3)
RRT patients (*n* = 19)	PopPK-Xie (15)	218.33	−56.19 ± 216.75	15.93 (−237.07 to 54.74)	24.07 ± 22.61	20.31 (6.32–30.66)
	ML model	197.11	−36.81 ± 198.95	−35.35 (−139.28 to 83.91)	22.25 ± 20.34	15.98 (7.11–35.60)
Infected patients (*n* = 20)	PopPK-Dvorchik (9)	216.33	113.74 ± 188.80	145.90 (90.51–240.29)	34.64 ± 19.14	35.08 (22.80–47.62)
	PopPK-Wu (14)	618.23	570.94 ± 243.30	605.21 (453.27–757.58)	105.54 ± 48.56	106.78 (76.58–139.57)
	PopPK-Balice (17)	273.13	133.45 ± 244.50	239.24 (19.75–285.35)	46.61 ± 33.81	44.74 (24.58–58.39)
	PopPK-Yamada (18)	448.22	388.71 ± 228.95	410.24 (314.70–523.38)	74.13 ± 35.86	78.91 (61.09–94.54)
	PopPK-Di Paolo (19)	209.49	22.29 ± 213.72	101.85 (−66.30 to 148.83)	29.30 ± 21.34	21.56 (16.23–39.32)
	Median PopPK	265.87	192.01 ± 188.69	248.90 (156.59–289.40)	48.56 ± 31.36	47.65 (26.33–58.39)
	Average PopPK	353.08	245.83 ± 223.85	300.49 (162.39–391.09)	58.05 ± 31.74	57.41 (40.26–75.89)
	ML model	141.45	96.48 ± 106.13	87.82 (32.73–167.72)	23.45 ± 21.28	13.99 (7.79–35.48)

^*a*^ RMSE, root mean square error; PE, prediction error; APE, absolute percentage error.

**TABLE 5:** Comparison of clinical scenarios with PopPK models

Patient group	Model	Prediction accuracy in “<666 mg·h/L”	Prediction accuracy in “666–939 mg·h/L”	Prediction accuracy in “>939 mg·h/L”	Overall accuracy
RRT patients (*n* = 19)	PopPK-Xie (15)	1/6 (16.7%)	5/7 (71.4%)	0/6 (0.0%)	6/19 (31.6%)
	ML model	3/6 (50.0%)	6/7 (85.7%)	1/6 (16.7%)	10/19 (52.6%)
	PopPK-Dvorchik (9)	7/14 (50.0%)	0/4 (0.0%)	2/2 (100.0%)	9/20 (45.0%)
Infected patients (*n* = 20)	PopPK-Wu (14)	0/14 (0.0%)	1/4 (25.0%)	2/2 (100.0%)	3/20 (15.0%)
	PopPK-Balice (17)	0/14 (0.0%)	3/4 (75.0%)	0/2 (0.0%)	3/20 (15.0%)
	PopPK-Yamada (18)	0/14 (0.0%)	1/4 (25.0%)	2/2 (100.0%)	3/20 (15.0%)
	PopPK-Di Paolo (19)	10/14 (71.4%)	2/4 (50.0%)	0/2 (0.0%)	12/20 (60.0%)
	Median PopPK	1/14 (7.1%)	1/4 (25.0%)	2/2 (100%)	4/20 (20%)
	Average PopPK	3.4/14 (24.3%)	1.4/4 (35%)	1.2/2 (60%)	6/20 (30%)
	ML model	11/14 (78.6%)	2/4 (50.0%)	2/2 (100.0%)	15/20 (75.0%)

#### Renal replacement therapy patients

The model produced a MAPE of 22.2% and an RMSE of 197 mg·h/L (Table 4). Prediction accuracies were 50.0% (<666 mg·h/L), 85.7% (666–939 mg·h/L), and 16.7% (>939 mg·h/L), with an overall accuracy of 52.6% (Table 5). Goodness-of-fit results of the real-world data validation are shown in Fig. 5.

### Comparison of prediction performance with PopPK model

Five infection PopPK models and one renal replacement therapy PopPK model were benchmarked against the ML approach.

#### Infected patients

Across the five PopPK models, the mean MAPE was 58.1%, and the mean RMSE was 353 mg·h/L. Using the ML model, the RMSE value decreased by 60.1% relative to the average of the PopPK models (relative decrease = [decrease in quantity ÷ original quantity] × 100%). The average PopPK model prediction accuracy of AUC_0–24_ in the range of “<666 mg·h/L”, “666–939 mg·h/L”, and “>939 mg·h/L” were 24.3%, 35.0%, and 60.0%, respectively. The average overall accuracy was 30.0%. Using the ML model, the overall accuracy increased by 150% relative to the average of PopPK models (relative increase = [increase in quantity ÷ original quantity] × 100%). The specific results for each model are shown in Tables 4 and 5.

#### Renal replacement therapy patients

The PopPK model yielded a MAPE of 24.1% and an RMSE of 218 mg·h/L. Using the ML model, the RMSE value decreased by 9.6% relative to the PopPK model. The PopPK model prediction accuracy of AUC_0–24_ in the range of “<666 mg·h/L”, “666–939 mg·h/L”, and “>939 mg·h/L” was 16.7%, 71.4%, and 0%, respectively. The overall accuracy was 31.6%. Using the ML model, the overall accuracy increased by 66.5% relative to the PopPK model. The specific results for each model are shown in Tables 4 and 5.

## DISCUSSION

In this study, we developed an ML model to predict daptomycin exposure (AUC_0–24_) across different disease states and demonstrated its strong predictive performance using real-world clinical data. Given the considerable variability in daptomycin pharmacokinetics under pathological conditions, this integrated approach provides a practical tool for optimizing initial dose selection in patient populations where conventional, disease-specific approaches are limited.

The final ML model identified dose, CRCL, dosing interval, body weight, and disease state as the most influential predictors of daptomycin exposure. As daptomycin is primarily renally eliminated, CRCL emerged as a critical determinant of drug disposition. Disease-specific effects were also captured: renal replacement therapy reduced systemic exposure, whereas renal transplantation increased it. These findings align with established pharmacokinetic knowledge, supporting the biological plausibility of the model outputs.

The training data were generated by integrating nine PopPK models from populations with various conditions: severe infections (17, 19, 22), MRSA infections (18), bone and joint infections (16), renal replacement therapy (6), renal transplant recipients (13), and renal impairment (20). A key strength of our integrated ML approach is its ability to recognize similarities among these diverse disease states and intelligently categorize them; for instance, three types of infections were aggregated into one group. The results indicate that ML is capable of analyzing complex data, integrating information from disparate sources, summarizing patterns, and enabling effective categorization.

Compared with traditional, stand-alone PopPK models, the ML model achieved notable improvements in predictive accuracy. For patients with infections, RMSE was reduced by 60.1% relative to the average of PopPK models. Even in renal replacement therapy patients, where PopPK models performed reasonably well, the ML model still achieved modest gains in RMSE and accuracy. These results underscore the potential of ML approaches to generalize across heterogeneous clinical scenarios.

The better predictive performance of the ML model in this study should be interpreted with caution. The model was trained on a large, simulated data set generated from several published PopPK models and therefore incorporated pharmacokinetic information from multiple clinical populations, disease states, covariate distributions, and dosing regimens. Its performance does not indicate that ML is inherently superior to PopPK modeling. PopPK models describe drug disposition using predefined compartmental structures and differential equations. When several disease states, dosing regimens, and covariates are considered together, it may be difficult to construct a single model that fully captures their combined and potentially nonlinear effects. ML models, in contrast, can learn these relationships directly from large heterogeneous data sets. In this study, this feature allowed information from different clinical settings to be combined for a priori estimation of AUC_0–24_. The proposed model is therefore better viewed as a tool for estimating exposure before TDM results are available, rather than as a substitute for PopPK-based precision dosing.

PopPK models also provide information that cannot be obtained from a single ML point estimate. By describing interindividual variability in pharmacokinetic parameters, they can generate a distribution or prediction interval for an individual AUC estimate and indicate the probability that exposure falls within the therapeutic target range. Their pharmacokinetic parameters and covariate effects are also clinically interpretable, which is useful for understanding sources of variability, evaluating alternative dosing regimens, and updating individual predictions when concentration data become available. In the present study, only point predictions from the PopPK models were used to allow a direct comparison with the deterministic outputs of the ML models. The comparison therefore focused on predictive accuracy and did not evaluate uncertainty quantification, mechanistic interpretation, or Bayesian updating. These remain important strengths of the PopPK framework.

Previous work by Woillard et al. also explored ML algorithms for daptomycin dose prediction and reported favorable accuracy in infection-related settings. Our study extends this concept by incorporating comorbid conditions, a clinically relevant challenge where pharmacokinetic alterations often complicate dosing. By validating the model with real-world patient data, we further demonstrated its translational potential in routine practice.

The advantages of ML over individual PopPK modeling lie in its flexibility and scalability (25, 26). Our approach can integrate diverse variables and detect complex, nonlinear relationships without predefined assumptions, whereas individual PopPK models require disease-specific structural equations and expert-driven simulations. This allows efficient adaptation to varied clinical scenarios and facilitates continuous refinement as new data become available.

**Fig 6:**
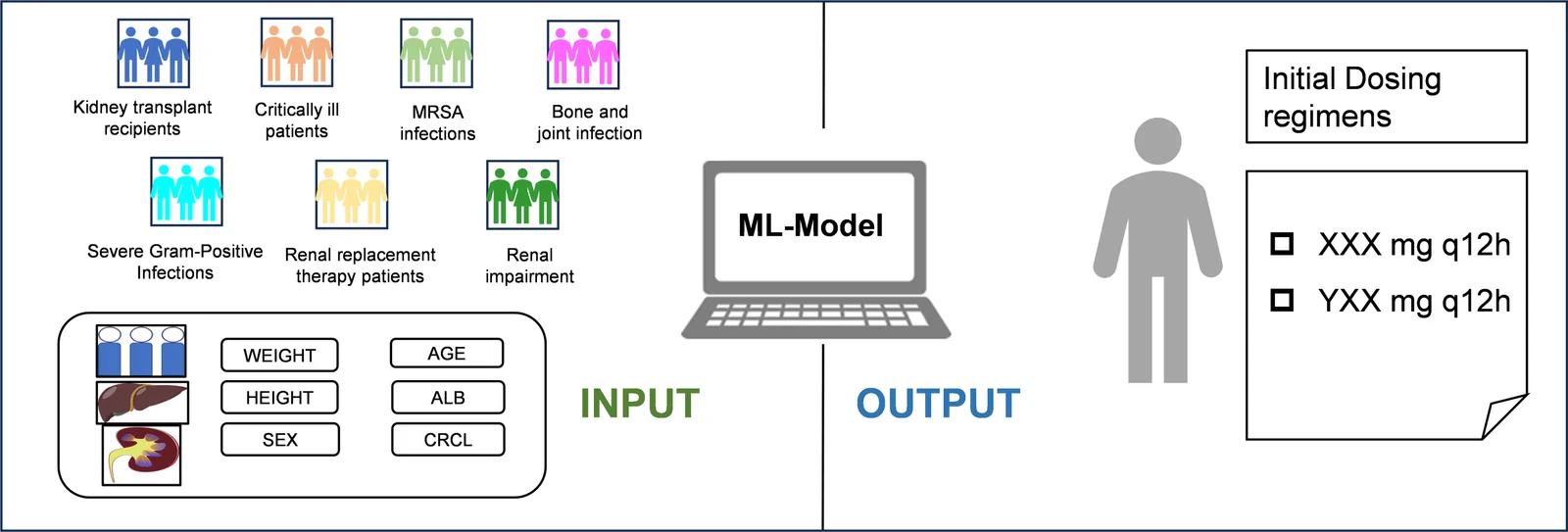
The application scenarios of the ML models.

In clinical practice, before initiation of therapy, the model integrates patient characteristics (e.g., comorbid disease state, dosing regimen, CRCL, body weight, ALB, sex, and age) to predict target AUC_0–24_ values, thereby guiding the selection of an individualized dose. A future objective is to incorporate these functions into a clinical decision-support platform, allowing seamless integration of pre-treatment dose selection into routine patient management (Fig. 6).

Nonetheless, limitations remain. First, the external validation data set included only patients receiving renal replacement therapy and infected patients. Thus, the validation was limited to these two populations and could not assess model performance in other subgroups, such as patients with different degrees of renal impairment, renal transplantation, or other disease conditions. Further validation of larger and more diverse data sets is needed. Second, simulated data sets generally exhibit lower variability than real-world cohorts, which may partially explain the discrepancy in predictive performance between simulation-based training and clinical validation. Third, the proposed ML model was not compared with a unified PopPK model developed from comparable individual-level data. The observed performance improvement of the ML model compared with PopPK models may partially reflect broader covariate coverage and integrated disease variability rather than intrinsic methodological superiority. Future work should aim to expand the data set to cover broader populations and to explore hybrid approaches that combine mechanistic PopPK modeling with ML methods.

In summary, our integrated ML framework represents a powerful complement to traditional pharmacometric approaches. Our results demonstrate that ML can achieve robust and clinically relevant predictions of daptomycin exposure, thereby providing a foundation for individualized dose optimization in patients with complex disease states. Translation of this model into a user-friendly clinical platform is expected to enhance precision dosing and improve therapeutic outcomes.
